# High day-to-day and diurnal variability of oxidative stress and inflammation biomarkers in people with type 2 diabetes mellitus and healthy individuals

**DOI:** 10.1080/13510002.2020.1795587

**Published:** 2020-07-21

**Authors:** Alistair R. Mallard, Siri Marte Hollekim-Strand, Charlotte Björk Ingul, Jeff S. Coombes

**Affiliations:** a Centre for Research on Exercise, Physical Activity and Health, School of Human Movement and Nutrition Sciences, The University of Queensland, Brisbane, Australia; b K. G. Jebsen Center for Exercise in Medicine at Department of Circulation and Medical Imaging, Norwegian University of Science and Technology (NTNU), Trondheim, Norway

**Keywords:** Type 2 diabetes, inflammation, oxidative stress, antioxidants, redox balance, variability, isoprostanes, protein carbonyls

## Abstract

**Objective:** Assess the variability and differences in oxidative stress, antioxidant, and inflammatory biomarkers in people with type 2 diabetes mellitus (T2D) and healthy controls.

**Methods:** Ten men and women diagnosed with T2D and ten healthy matched controls (CON) were recruited. Participants had venous blood taken at six different time points on different days, three in the morning (after overnight fast) and three in the afternoon. Inflammation (IL-6, 8, 10 and TNF-α), oxidative stress/antioxidant biomarkers (F_2_-isoprostanes, protein carbonyls, total antioxidant capacity (TAC), glutathione peroxidase activity, IL-6, 8 & 10 and TNF-α) were assessed.

**Results:** Biomarker concentrations were similar between groups. There was large variability in nearly all biomarkers for both groups. For inflammatory measures, intra-individual coefficients of variation (CV) ranged from 64.0–92.1% and 100.9–259.0% for inter-individual differences. CVs for oxidative stress markers were lower (7.4–31.2% for intra-individual and 8.6–43.0% for inter-individual). TAC had the lowest intra-individual CV – 7% for T2D and 8% for CON. Protein carbonyls were more variable in the afternoon (34% CV) compared to morning (24% CV) in CON. IL-6 intra-individual CV was different between groups for afternoon measurements (93% T2D, 60% CON).

**Conclusion:** Oxidative stress and inflammatory biomarkers show considerable variation in both T2D and healthy populations.

**Trial registration:** ClinicalTrials.gov identifier: NCT01206725.

## Introduction

Oxidative stress and inflammation share a cyclical link and are implicated in the pathogenesis of T2D [1,2]. Oxidative stress is a disturbance in the balance between pro- and anti-oxidants which can lead to disrupted cellular signalling and cellular damage [3]. Unchecked highly reactive pro-oxidants damage cellular lipids, proteins and modify deoxyribonucleic acid [4–6]. Subsequently, continual oxidative stress and disturbance to regular cellular redox homeostasis are associated with chronic inflammatory responses; as evidenced in many long-term illnesses, such as T2D [7]. These findings have led to many studies investigating the root causes of oxidative stress, inflammation and the pathogenesis of T2D [8].

Measurement of pro- and antioxidants has been a topic of debate amongst the literature for decades [9–11]. Several previously accepted biomarkers of oxidative stress have been shown to be inaccurate or unreliable [11,12]. The gold standard for measuring lipid peroxidation *in vivo*; F_2_-isoprostanes, is contentious amongst scholars due to its reliability in determining lipid peroxidation without careful sample preparation and use of expensive complex instrumentation, such as gas chromatography mass spectrometry[5]. Protein carbonyls are recognised as the gold-standard measurement of protein oxidation, however, there is debate as to whether protein carbonyls are a true measure of protein oxidation, or if protein carbonyls are also involved in antioxidant defence as signalling molecules [13].

Knowledge of biomarker variability is crucial to determining perturbations from technical errors and biological noise [14]. A greater signal to noise ratio for any measurement allows for improved accuracy and precision when determining causal or correlation relationships [14,15]. The same can be said for oxidative stress and inflammatory biomarkers, if inter- and intra-individual variability is high it becomes harder to distinguish real change from natural perturbations and cyclical rhythms, such as dietary modifications or diurnal variability [16]. An ideal biomarker is one that is stable to all manner of internal and external factors bar that of the factor of interest e.g. a one factor system. However, due to the complex nature of human physiology a one factor biomarker is likely to be a fruitless pursuit. Determining the background noise for current biomarkers of pro/antioxidants and inflammatory biomarkers is essential in clinical decision making (e.g. beginning antioxidant therapy) and clinical trial sample size determination [17].

Indeed, there is limited data on variability in oxidative stress and inflammatory biomarkers in healthy populations and people with T2D. One previous study conducted sampled urine daily over a one-month period in 19 healthy individuals [16]. Intra-individual coefficients of variation for oxidative stress biomarkers ranged from 29% (8-hydroxy-2′-deoxyguanosine) to 149% (F_2_-isoprostane isomer – 15(R)-prostaglandinF_2α_). We are unaware of any studies measuring oxidative stress biomarker variability in people with T2D. Therefore, the aims of this study were, in healthy individuals and people with T2D, to [1] assess the variability (day-to-day and diurnal) of oxidative stress and inflammatory biomarkers and [2] investigate differences between groups in these measures.

## Methods

This is a sub-study of a randomised, controlled trial [18]. Ten participants with T2D and ten healthy matched controls completed the study. The protocol was approved by the Regional Committee for Medical and Health Research Ethics of Central Norway and was registered with the Clinical Trials Registry (ClinicalTrials.gov identifier: NCT01206725). Written informed consent was obtained from all participants prior to baseline testing and they were insured through the intervention period.

Inclusion criteria for the T2D participants were diagnosis of T2D less than ten years ago, non-insulin dependent, aged between 40 and 65 years, and achieving less than 210 min/week of exercise. Exclusion criteria included known cardiovascular or lung disease, uncontrolled hypertension, orthopaedic or neurological restrictions, body mass index (BMI) >35 kg/m^2^, pregnancy, drug, tobacco or alcohol abuse, planned surgery during the trial period, serious eating- and/or personality disorders, and reluctance to sign informed consent form. Participants were recruited through local newspaper advertising and from the outpatient population at St. Olav’s University Hospital, Trondheim, Norway.

Upon arriving at the laboratory in the afternoon, participants height and weight were measured, and a blood sample drawn from an antecubital vein. Participants were asked to avoid strenuous exercise for 24-hours prior to testing. Each participant was then asked to return to the laboratory for blood collection a further 5 times. Two afternoon collections and three morning collections on separate days over a two-week period.

Ethylenediaminetetraacetic acid vacutainers (Vacuette, Greiner Bio-One, Belgium) were used to collect all blood samples. Samples were immediately stored on ice before being centrifuged at 3000 rpm for 10 min at 4 degrees Celsius. Separated plasma was then aliquoted and stored at −80°C until biochemical assays were performed. Samples were shipped in dry ice to the University of Queensland, Brisbane, Australia for inflammatory and oxidative stress biomarker analysis.

Plasma isoprostanes were analysed via gas chromatography mass spectrometry (Varian, Belrose, Australia) as we have previously described in Briskey, Wilson [19]. Briefly, isoprostanes were extracted from plasma after saponification with methanolic NaOH. 8-iso-PGF2α-d4 (Cayman Chemicals, Ann Arbor, MI) was used as an internal standard and added to samples before incubation at 42°C for 60 min. Samples were acidified to pH 3 with hydrochloric acid and hexane was added before 10 min of centrifugation. The supernatant was removed, and the remaining solution extracted with ethyl acetate before being dried under nitrogen. Acetonitrile was used to reconstitute samples prior to drying in silanized glass inserts. Derivatization with pentafluorobenzylbromide and diisoproplyethylamine followed. Incubation for 30 min at room temperature preceded drying samples under nitrogen. Pyridine, bis(trimethylsilyl)trifluoroacetamide 99% and trimethylchlorosilane 1% were added and samples were incubated at 45°C for 20 min. Finally, hexane was added, and samples were mixed before analysis.

Plasma protein carbonyls were analysed using an adaption of the Levine, Garland [20] method as we have previously described in Mullins, van Rosendal [21]. Samples were incubated with 2,4 dinitrophenylhydrazine in 2.5 M HCl for 1 h in the dark. Plasma blanks were incubated in 2.5 M HCl only. Samples were precipitated with 20% trichloroacetic acid (TCA) on ice and centrifuged at 10,000 g for 10 min. Supernatants were discarded, and the pellets resuspended in 10% TCA and centrifuged as above. Supernatants were removed and the pellets resuspended in 1:1 ethanol:ethylacetate solution. After centrifugation as above, the pellets were washed twice more in ethanol:ethylacetate solution. Pellets were then resuspended in 6 M guanidinehydrocholride solution and 220 μL of samples and blanks were transferred to microplate wells and absorbance read at 370 nm with correction at 650 nm using a microplate reader (Fluostar Optima, BMG Labtech, Offenburg, Germany). PC concentration was normalised to plasma protein content measured using a Pierce BCA protein assay kit (Thermo Scientific, Victoria, Australia).

Plasma glutathione peroxidase activity was measured using an adaptation of the method from Wheeler et al. Glutathione peroxidase activity was measured as the rate of oxidation of nicotinamide adenine dinucleotide phosphate, reduced (NADPH) at 340 nm in a coupled reaction, cycling oxidised glutathione to reduced glutathione using glutathione reductase. The reduced glutathione is utilised by glutathione peroxidase when the reaction is started with the addition of t-butyl hydroperoxide. These measures were performed on an auto analyser (Cobas Mira, Roche Diagnostica, Basel, Switzerland).

Total antioxidant capacity (TAC) was measured using a modified version of an assay previously described, 20 and adapted for an auto analyser (Cobas Mira, Roche Diagnostica, Switzerland). Plasma was incubated with metmyoglobin and 2,2′-azino-biz (3-ethylbenzothiazoline-6-sulphonic acid). Post incubation hydrogen peroxide was added, and the sample was again incubated. Absorbance was measured spectrophotometrically to determine TAC.

Plasma cytokines (interleukins 6, 8, 10 and tumour necrosis factor-alpha) were measured using the Milliplex MAP Human High Sensitivity T Cell Magnetic Bead Panel Kit. The assay protocol was followed as set out by Millipore, and magnetic beads were analysed by a multiplex analyser (Athena Multi-lyte, Zeus Scientific, Raritan, NJ, USA).

Blood glucose, C-peptide, plasma triglycerides, total cholesterol, high density lipoprotein and low-density lipoprotein, high-sensitive C-reactive protein and HbA1c were analysed as previously reported in Hollekim-Strand, Malmo [18]. Insulin sensitivity was calculated using the HOMA2 calculator (The Homeostasis Assessment Model, University of Oxford, UK).

Laboratory coefficients of variation (CV) for inflammatory and oxidative stress biomarkers have previously been developed by assaying 20 replicate samples. Laboratory CV’s are as follows; F2-isoprostanes – 7%, protein carbonyls – 11.9%, glutathione peroxidase activity – 2.4%, total antioxidant capacity – 1.9%, plasma cytokines – 10%.

All data analyses were completed using SPSS software (version 25 for Windows, SPSS Inc., Chicago, IL). Normality testing of data using the Shapiro–Wilk test was performed. Between group differences were calculated using *t*-tests or Mann–Whitney *U* tests, where appropriate. The coefficient of variation (CV) is defined as the ratio of the standard deviation to the mean of a sample. CV was calculated as a measure of biomarker variability from the mean and determined the between- and within-individual (within each group) variability. Intraclass correlation coefficients (ICC) are used to describe the relationship between participants in each group and were calculated using log transformed within and between group standard deviations to determine the reliability of biomarkers [22]. Guidelines for interpretation of ICC values are as follows: <0.50 – poor, 0.50–0.75 – moderate, 0.76–0.90 – good, >0.90 – excellent [23]. Sample sizes were calculated using the retest correlation coefficient and our laboratory analytical variability; $n=(200\times (1-ICC))\times CV_{\left(biomarker\right)}$ [22]. All data are represented as mean ± SD or median (IQR), and significance was assumed when *p* < 0.05.

## Results

Participant characteristics for the patients with type 2 diabetes and control groups can be seen in Table 1. As expected, T2D had higher HbA1c and fasting glucose above that of CON (*p* < 0.05), however, groups were matched for all other characteristics.

**Table 1.** Participant characteristics.

	CON (*n* = 10)	T2D (*n* = 10)
Diabetes duration (years)	–	3.1 ± 1.8*
Female (*n*, %)	3 (30%)	3 (30%)
Age (years)	52.8 ± 10.1	53.4 ± 8.1
Height (cm)	1.79 ± 0.09	1.76 ± 0.09
Weight (m)	92.9 ± 19.1	87.7 ± 15.2
BMI (kg/m^2^)	28.5 ± 4.2	28.3 ± 3.8
Waist girth (cm)	104.4 ± 13.8	107.1 ± 27.5
HbA1c (%)	5.46 ± 0.22	6.35 ± 0.97*
Glucose (mmol/L)	5.26 ± 0.47	7.28 ± 1.84*
HOMA-IR	1.7 ± 0.7	2.2 ± 0.7
Cholesterol (mmol/L)	5.04 ± 0.53	5.04 ± 1.08
Triglycerides (mmol/L)	1.25 ± 0.57	1.90 ± 1.12
HDL (mmol/L)	1.40 ± 0.48	1.24 ± 0.36
LDL (mmol/L)	3.08 ± 0.42	2.95 ± 1.04
Hb (g/dl)	14.5 ± 0.9	14.7 ± 1.3
hs-CRP	1.28 ± 0.90	2.51 ± 2.70

NB. * denotes difference between T2D and CON (*p* < 0.05). BMI – body mass index, HbA1c – glycated haemoglobin, HOMA-IR – homeostatic model assessment – insulin resistance, HDL – high density lipoprotein cholesterol, LDL – low density lipoprotein cholesterol, Hb – haemoglobin, hs-CRP – high sensitivity C-reactive protein.

**Figure 1.**
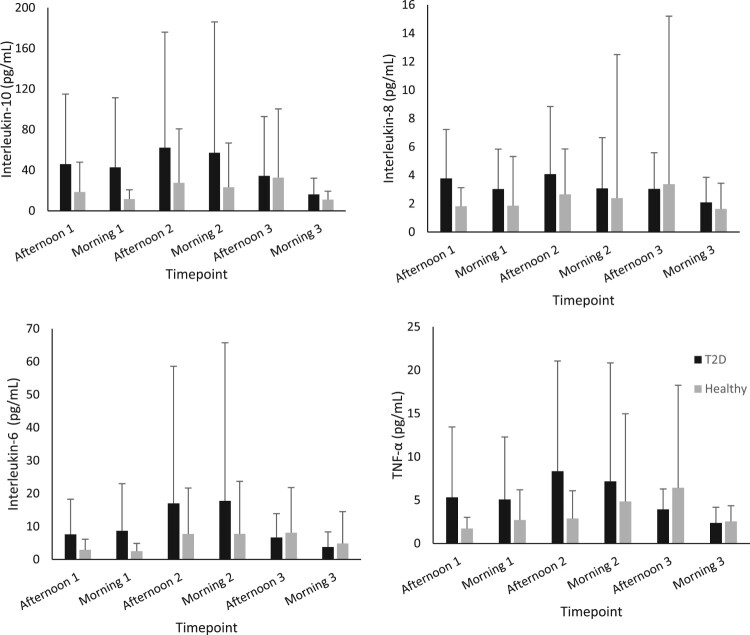
Concentration of plasma inflammatory biomarkers in participants with type 2 diabetes and healthy control (mean ± SD)*. N.B.* TNF-α – Tumour necrosis factor alpha.

**Figure 2.**
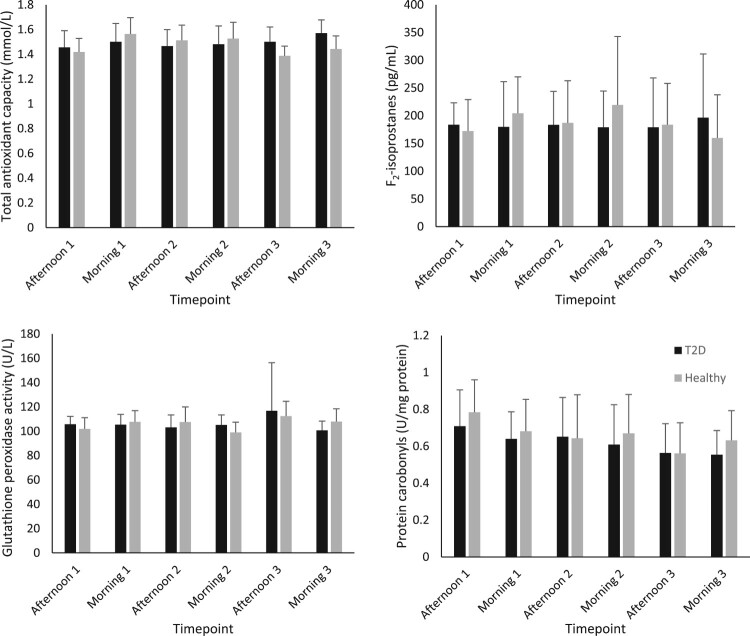
Concentration of plasma oxidative stress biomarkers in participants with type 2 diabetes and healthy control (mean ± SD).

Figures 1 and 2 show the group average concentrations of inflammatory and oxidative stress biomarkers at each visit. Large standard deviations were noted for all inflammatory markers (IL-6, 8, 10 and TNF-α). The median and interquartile range for each biomarker is displayed in Table 2. Interestingly the inflammatory biomarkers displayed a much higher IQR to median ratio compared to oxidative stress biomarkers. All the biomarkers of inflammation had IQR’s that were larger than the median value indicating large variabilities in the measures. The biomarker with the smallest IQR was glutathione peroxidase activity.

**Table 2.** Median (IQR) and intraclass correlation coefficient (90% CI) for inflammatory and oxidative stress biomarkers.

	Median (IQR)		Intraclass correlation coefficient (90% CI)	
	T2D	CON	T2D	CON
Inflammation				
Interleukin-10 (pg/mL)	14.78 (24.24)	8.96 (9.04)	0.53 (0.29–0.78)	0.20 (0.00–0.52)
Interleukin-8 (pg/mL)	2.04 (2.05)	1.45 (1.15)	0.41 (0.18–0.70)	0.53 (0.29–0.78)
Interleukin-6 (pg/mL)	2.93 (6.67)	1.88 (1.65)	0.46 (0.22–0.73)	0.64 (0.42–0.85)
Tumour necrosis factor-α (pg/mL)	3.03 (3.27)	1.96 (2.24)	0.39 (0.16–0.69)	0.32 (0.09–0.63)
Oxidative stress				
F_2_-isoprostane (pg/mL)	171.34 (71.22)	173.15 (96.94)	0.19 (0.00–0.52)	0.40 (0.17–0.69)
Glutathione peroxidase activity (U/L)	106.65 (10.01)	105.20 (9.37)	0.47 (0.22–0.75)	–
Total antioxidant capacity (mmol/L)	1.50 (0.25)	1.46 (0.26)	0.19 (0.00–0.51)	0.01 (−0.12–0.28)
Protein carbonyls (U/mg protein)	0.61 (0.28)	0.66 (0.23)	–	–

Table 3 provides the intra-individual CV for each biomarker during morning/afternoon visits, or all timepoints combined. For all time-points combined, the CV for the inflammatory measures ranged from 64.0–92.1% and 7.4–31.2% for the oxidative stress/antioxidant markers. The only biomarker that showed differences in variability between groups was IL-6 with the CV for CON 33% lower during afternoon visits than for T2D (*p* < 0.05). Diurnal differences for PC in CON were noted, with the CV in the morning 11% less than the afternoon sessions (*p* < 0.05). There were no between group differences in intra-individual biomarker CV when all timepoints were pooled together.

**Table 3.** Average intra-individual coefficients of variation (CV) for inflammatory and oxidative stress biomarkers (%).

	Combined (average of 6 timepoints)		Afternoon (average of 3 timepoints)		Morning (average of 3 timepoints)	
	T2D	CON	T2D	CON	T2D	CON
Interleukin-10	77.7	64.0	79.7	52.3	84.1	63.7
Interleukin-6	92.1	66.9	93.4*	60.1*	79.9	59.9
Interleukin-8	62.8	55.3	64.4	50.0	67.8	53.6
Tumour necrosis factor-α	79.6	78.4	82.8	63.1	76.2	73.6
F_2_-isoprostanes	31.2	29.7	28.0	24.9	32.8	29.9
Glutathione peroxidase activity	10.9	9.3	9.7	8.5	6.6	8.6
Total antioxidant capacity	7.4	8.7	7.1	6.4	7.4	8.3
Protein carbonyls	27.4	28.5	30.3	34.2†	27.8	23.7†

N.B. * denotes a difference between groups, † denotes a diurnal difference.

Inter-individual variability is shown in Table 4. There were no group or diurnal differences in CVs. The inflammatory markers had very large variabilities, for all time points combined they ranged from 100.6 to 259% compared to the oxidative stress/antioxidant measures; 8.6to 43.0%.

**Table 4.** Average inter-individual coefficients of variation (CV) for inflammatory and oxidative stress biomarkers (%).

	Combined (average of 6 timepoints)		Afternoon (average of 3 timepoints)		Morning (average of 3 timepoints)	
	T2D	CON	T2D	CON	T2D	CON
Interleukin-10	190.4	195.1	167.7	186.3	161.5	114.1
Interleukin-6	259.0	194.4	164.6	154.5	186.0	167.1
Interleukin-8	100.9	143.7	97.2	134.6	98.0	102.0
Tumour necrosis factor-α	162.7	187.8	121.4	123.8	136.4	135.8
F_2_-isoprostanes	41.1	43.0	34.7	38.0	46.7	45.7
Glutathione peroxidase activity	17.1	10.2	16.6	10.4	7.8	8.9
Total antioxidant capacity	8.8	8.6	8.8	7.2	8.9	8.1
Protein carbonyls	29.1	29.1	29.5	29.5	27.3	27.4

The intraclass correlation coefficient (ICC) was calculated as a reliability measure for each biomarker (Table 2). No biomarkers could be classed as ‘excellent’ or ‘good’ in relation to reliability. Interleukin-10 seems to be the most reliable biomarker for people with T2D (ICC – 0.53), whilst IL-8 and IL-6 are the most reliable for the control group with ICC’s of 0.53 and 0.64, respectively. All other biomarkers are classed as having ‘low’ reliability with ICC’s of less than 0.50. Some ICC’s could not be calculated (PC/GPx) most likely due to large differences between intra- and inter-individual variability.

## Discussion

The aims of this study were to determine the day-to-day and diurnal variability, concentration and, consequently, reliability of inflammatory and oxidative stress biomarkers in people with type 2 diabetes and healthy controls. Our data show large between and within individual differences in almost all oxidative stress and inflammatory biomarkers for both healthy controls and people with T2D. These very high CV’s have considerable implications for their use in diagnosis, clinical decision making and research. Distinguishing a change in oxidative stress or inflammatory biomarker from ‘normal’ variation would be challenging at best.

Interestingly, we found little difference in both the variability and the concentration of oxidative stress and inflammatory biomarkers between people with T2D and healthy controls. This is in contrast to much of the current literature suggesting that T2D pathogenesis is correlated with chronic low-grade inflammation and, by its cyclical relationship, also oxidative stress [24]. We did find that people with T2D have a larger within-individual variability for IL-6 during the afternoon than that of control participants (93% vs 60%). Secondary to these findings, also unanticipated, was a diurnal difference in within-individual variability for protein carbonyls in the control group (34% afternoon vs 24% morning).

Previous research into oxidative stress biomarker variability by our group has reported similar within and between individual CV’s in F2-isoprostanes and protein carbonyls to this study [17]. The variability findings by Dahwa, Fassett [17] were consistent with previous oxidative stress biomarker variability research, however the study population was haemodialysis patients with a ∼2-fold higher concentration of F_2_-isoprostanes compared to this study cohort. Whilst the variability between individuals may be large this could be explained by biological individuality as previously discussed by Margaritelis, Theodorou [25].

ICC’s closer to 1 indicate that whilst there may be large between individual variability the within-individual variability is small, which is indicative of a more reliable biomarker. Contrary to expectations, this study did not find high ICC’s for any biomarkers measured (Table 2). Indeed, the variability of some biomarkers was so large that ICC’s could not be calculated.

Using our analytical variability and the ICC we calculated the sample sizes needed for a two-group research study to detect a low effect size (*d* = 0.2) with our inflammatory and oxidative stress markers (Table 5). These sample sizes indicate that small studies would likely be unable to detect real differences (type 2 errors), assuming the variability we found was normal. At least, investigators should know the variability of their measures within their population using their analytical approaches.

**Table 5.** Number of participants (n) needed per group to obtain a low (*d *=* *0.2) effect size for a randomized control trial or crossover trial design in people with T2D.

	Randomized control design	Crossover design
Interleukin-10 (pg/mL)	104	26
Interleukin-8 (pg/mL)	131	33
Interleukin-6 (pg/mL)	120	30
Tumour necrosis factor-α (pg/mL)	136	34
F_2_-isoprostane (pg/mL)	180	45
Glutathione peroxidase activity (U/L)	118	30
Total antioxidant capacity (mmol/L)	180	45

N.B. Sample size is calculated using the following assumptions: *α *=* *0.05, power* *=* *0.8, allocation ratio of 1:1, two-tailed difference between means, effect size *d *=* *0.2.

External factors affecting plasma concentration of oxidative stress and inflammation markers are difficult to control in any population. Previous research has suggested that diet, exercise, sleep, and mood all impact the concentration of oxidative stress and inflammatory markers [26]. Whilst this study attempted to address these issues by having blood draws performed at the same time, with an overnight fast for morning sessions, there is a portion of the variability which could be explained by several external factors. Under an extremely controlled environment we would expect the variability of biomarkers to decrease, however, the applicability of such research to real world scenarios would be limited. Analytical variability also contributes to the variation in collected sample analyses, however, our laboratory has previously reported low CV’s (between 2 and 12%) for the inflammatory and oxidative stress biomarkers measured [19,21,27].

The individual variability in oxidative stress and inflammatory biomarkers is interesting and certainly warrants further investigation. Future studies would benefit from assessing a multitude of areas likely to contribute to biomarker variability, including; nutrition, mood, exercise status, as well as general health markers. A long-term study (6+ months) assessing an increased sample size may provide further information towards determining whether reference ranges can be set for oxidative stress and inflammatory biomarkers.

In summary, we report that people with T2D have similar variability of oxidative stress and inflammatory biomarkers to that of their matched controls. Interleukin-6 shows considerably more variability in people with type 2 diabetes, however this was found to be only in samples taken in the afternoon suggesting that there may be some diurnal variation in people with type 2 diabetes. Considering the variability observed in inflammatory and oxidative stress biomarkers in people with type 2 diabetes we recommend careful consideration of the sample size needed to detect both the minimal detectable difference between populations and clinically relevant results before commencing any further research. We caution the use of oxidative stress and inflammatory biomarkers in any clinical decision making for a type 2 diabetic population due to the high variability and poor reliability.
